# Histological guided treatment in paediatric patients presenting with severe left ventricular dysfunction

**DOI:** 10.1007/s00431-025-06424-x

**Published:** 2025-09-18

**Authors:** Paola Dolader, Diana Carolina Juzga Corrales, Roger Esmel-Vilomara, Carla Daina, Jaume Izquierdo-Blasco, Eva Fernandez, Marta Garrido-Pontnou, Alexandra Navarro, Jessica Camacho, Andrea Fidalgo, Rebeca Sánchez-Salmador, Fuensanta Escudero, Moisés Sorli, Pedro Betrian-Blasco, Ferran Roses-Noguer, Ferran Gran

**Affiliations:** 1 Faculty of Medicine, Universitat Autònoma de Barcelona, Barcelona, Spain; 2 Paediatric Cardiology, Universitat Autònoma de Barcelona, Vall d’Hebron Hospital Campus, Barcelona, Spain; 3 Paediatric Cardiology, Hospital de La Santa Creu I Sant Pau, Barcelona, Spain; 4 Paediatric Intensive Care, Vall d’Hebron Hospital Campus, Barcelona, Spain; 5 Pathological Anatomy, Vall d’Hebron Hospital Campus, Barcelona, Spain; 6 Paediatric Cardiology, Hospital Universitario Son Espases, Palma, Spain; 7 Paediatric Cardiology, Hospital Clínico Universitario Virgen de La Arrixaca, Murcia, Spain

**Keywords:** Dilated cardiomyopathy, Myocarditis, Endomyocardial biopsy, ParvovirusB19

## Abstract

Differential diagnosis in children presenting with dilated cardiomyopathy and severe cardiac dysfunction is challenging. In our hospital, a protocol was established in 2015 in which endomyocardial biopsy (EMB) was performed in selected patients and treatment was guided by biopsy results. The study aims to describe our experience with this diagnostic and therapeutic strategy. We performed a unicenter paediatric ambispective study that include patients with dilated cardiomyopathy and severe cardiac dysfunction (left ventricular ejection fraction (LVEF) < 35%) in whom EMB was performed and treatment was prescribed based on EMB results from February 2015 to December 2024. 23 patients (24 episodes) were included. 15 patients had a complete recovery, 5 patients had a partial response to treatment and 4 had no response to treatment. Patients were divided into two groups, those with complete recovery (15) and those without complete recovery (9). No differences were observed in sex, age, clinical presentation, need for Extracorporeal Membrane Oxygenation (ECMO) or echocardiogram parameters. Complete recovery was associated with a higher degree of inflammation on EMB (*p* < 0.001), necrosis (= 0.007) and oedema (*p* = 0.036), negative genetic testing (*p* < 0.001), higher troponin values (*p* = 0.015) and positive viral PCR in myocardial tissue (*p* < 0.001). *Conclusion*: EMB is a valuable tool for diagnosis and treatment of paediatric patients presenting with dilated cardiomyopathy. Factors associated with a favourable response to therapy include: intense inflammatory infiltrate in EMB, oedema, necrosis, positive viral PCR found in the myocardium, high elevation of troponins and negative genetic testing.

**Table:** 

**What is Known:**
• *Differential diagnosis in paediatric patients presenting with severe dysfunction is challenging* • *EMB remains the gold standard technique for the diagnosis of myocarditis and can help to guide treatment*
**What is New:**
• *EMB guided treatment my benefit paediatric patients with inflammatory cardiomyopathy*. • *Factors associated with a favourable response to treatment in patients presenting with severe cardiac dysfunction include: intense inflammatory infiltrate in EMB, oedema, necrosis, positive viral PCR in the myocardium, high elevation of troponins and negative genetic testing*. • *Positive genetic testing was observed in some patients with inflammatory cardiomyopathy. In our opinion, genetic testing should be considered in all patients presenting with severe dysfunction*. • *Interferon β combined with corticosteroids may offer a beneficial treatment approach in patients with Parvovirus B19-induced myocarditis*

• *Differential diagnosis in paediatric patients presenting with severe dysfunction is challenging*

• *EMB remains the gold standard technique for the diagnosis of myocarditis and can help to guide treatment*

• *EMB guided treatment my benefit paediatric patients with inflammatory cardiomyopathy*.

• *Factors associated with a favourable response to treatment in patients presenting with severe cardiac dysfunction include: intense inflammatory infiltrate in EMB, oedema, necrosis, positive viral PCR in the myocardium, high elevation of troponins and negative genetic testing*.

• *Positive genetic testing was observed in some patients with inflammatory cardiomyopathy. In our opinion, genetic testing should be considered in all patients presenting with severe dysfunction*.

• *Interferon β combined with corticosteroids may offer a beneficial treatment approach in patients with Parvovirus B19-induced myocarditis*

## Introduction

Dilated cardiomyopathy (DCM) is a severe disease with a relatively low incidence at 3–6 cases per 100,000 children but with high morbidity and mortality [1]. DCM can have different causes, including genetic cardiomyopathy and myocarditis.

Myocarditis is an inflammation of the myocardium caused mainly by viral infection. Parvovirus B19 (PVB19) is one of the leading causes in paediatric patients [2]. The presentation varies widely from acute presentation with nonspecific symptoms to a severe ventricular dysfunction and cardiogenic shock or subacute infection that can cause chronic inflammation with ventricular dilatation and dysfunction.

Endomyocardial biopsy (EMB) remains the gold standard technique for diagnosing myocarditis. Although it is an invasive procedure that is not exempt from risks, EMB provides histological confirmation and can help guide treatment [3, 4]. The addition of immunohistochemistry, which enables the detection of subtle inflammatory infiltrates, and viral polymerase chain reaction (PCR) has significantly improved the diagnostic sensitivity of EMB.

Some studies suggest that the presence of pathogenic or likely-pathogenic variants in cardiomyopathy-related genes may increase susceptibility to developing myocarditis [5, 6]. Conversely, bursts of active myocardial inflammation have been reported in patients with known genetic cardiomyopathies [7]. Furthermore, chronic inflammation can also be observed in patients with genetic DCM, as a consequence of the inflammatory cascade associated with heart failure [8–10]. These findings point to a potential overlap between the inflammation associated with heart failure and that observed in histologically confirmed or “borderline” myocarditis. Consequently, despite the challenge of achieving a definitive diagnosis (particularly in paediatric patients with inflammatory DCM), establishing an accurate diagnosis is crucial for both prognosis and therapeutic decision-making. Treatment targeting myocardial inflammation and viral infection has been evaluated and proposed by several authors [11–15].

**Fig. 1:**
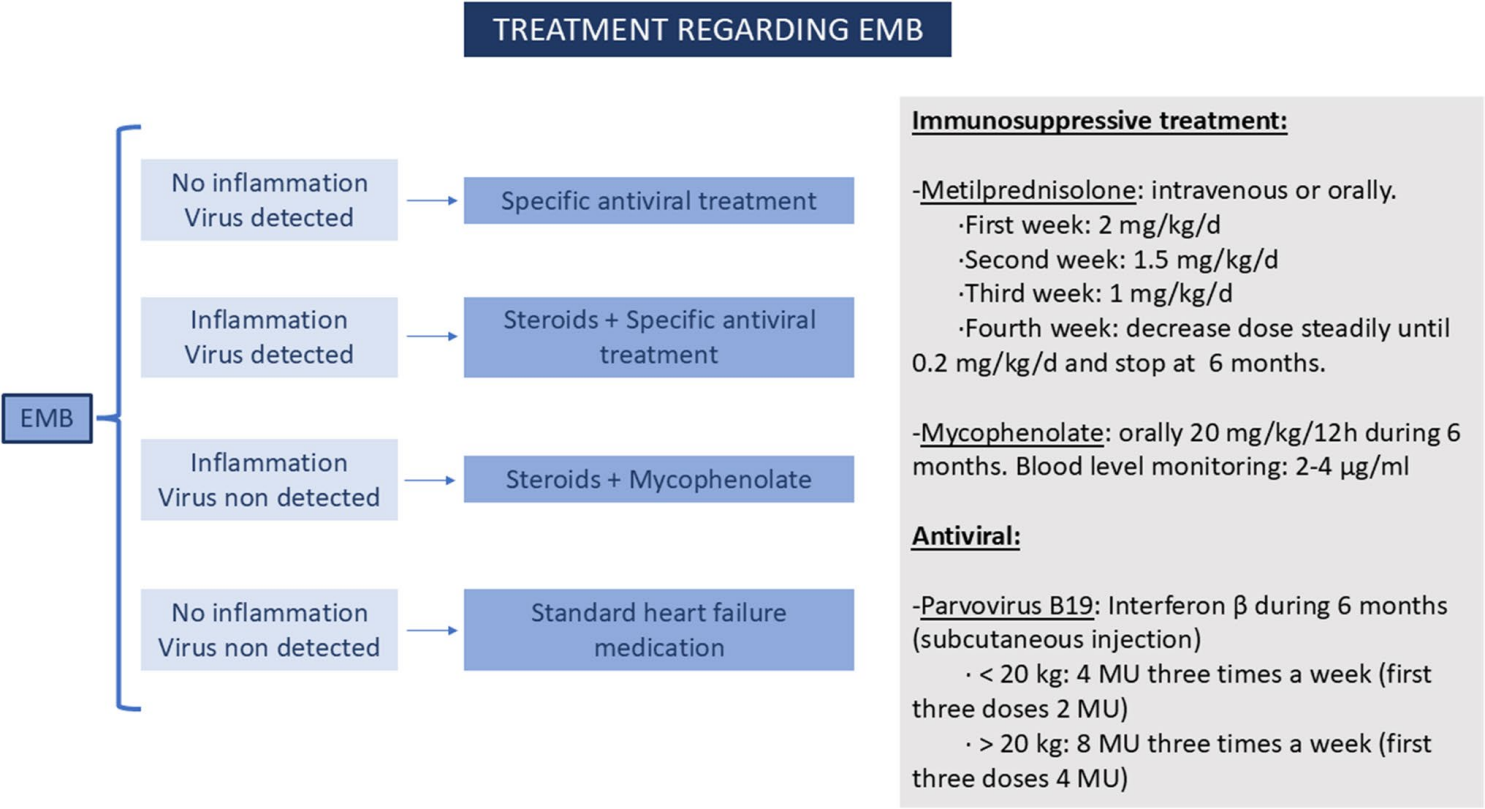
Treatment protocol

In our hospital, a protocol for diagnosing and treating myocarditis and inflammatory cardiomyopathy was established in 2015 (Fig. 1). The present study aims to review retrospectively and prospectively paediatric patients admitted to our hospital between February 2015 and December 2024 with DCM and severe left ventricular dysfunction (defined as a left ventricular ejection fraction (LVEF) < 35%) in whom myocardial inflammation was demonstrated by EMB, thereby establishing a diagnosis of acute myocarditis or inflammatory cardiomyopathy in accordance with the 2013 position statement of the European Society of Cardiology [4]. Treatment was indicated depending on immunohistology and microbiologic findings.

Data from some of the patients in this study have been previously published in two separate publications. The first study [16] aimed to identify clinical presentation patterns and assess invasive and non-invasive measures for differentiating acute myocarditis (with or without left ventricular dilatation) from genetic cardiomyopathy. Most participants in that previous work were enrolled before the implementation of our current protocol and therefore did not receive specific treatment based on EMB findings. The second study describes our experience with Interferon β (IFN-β) and steroid therapy in patients with confirmed PVB19-myocarditis [17].

## Material and methods

### Patient population

The study was conducted at the Paediatric Cardiology Department at Vall d’Hebron University Hospital. We included patients that were admitted in our centre younger than 18 years old presenting with a new-onset left ventricular dilation of unknown origin (defined as left ventricular end-diastolic diameter (LVEDD) z-score >  + 2) and severe left ventricular dysfunction (LVEF < 35%) in whom EMB demonstrated myocardial inflammation and treatment was prescribed regarding EMB results. Some patients were referred from other hospitals in Spain.

### Vall d’Hebron protocol for paediatric patients

Following our protocol, generic blood tests with troponins and N-terminal pro-brain natriuretic peptides (NT-proBNP) and microbiologic tests with blood PCR for cardiotropic virus were performed at admission [4, 18]. Genetic testing was performed in all patients, using the TruSight Cardio Panel (Next Generation Sequencing including exonic and flanking intronic regions for 175 cardiomyopathy-associated genes). Variant classification was performed according to the guidelines established by the American College of Medical Genetics and Genomics [19].

According to our established protocol, EMB is considered in paediatric patients older than 6 months of age or weighing ≥ 7 kg with suspected myocarditis, who meet one of the following criteria:

- Cardiogenic shock requiring venoarterial Extracorporeal Membrane Oxygenation (ECMO).
- LVEF < 35% with hemodynamic compromise without echocardiographic improvement after ≥ 1 week of medical treatment.
- LVEF < 35%, with hemodynamic stability showing no significant improvement after ≥ 2 weeks of medical treatment.

EMB was performed through jugular access with a 6Fr bioptome, and 6 samples were obtained from the right interventricular septum: 4 were sent to pathology and 2 to microbiology to investigate cardiotropic virus described in the literature [4, 15]. Haematoxylin–eosin, Mason’s trichrome, and immunohistochemical stains for CD45, CD20, CD3, and CD68 were performed. Myocardial inflammation was defined following the Marburg immunohistological criteria of ≥ 14 mononuclear cells with ≥ 7 CD3 lymphocytes per mm^2^ [4].

All patients received standard heart failure therapy if haemodynamically stable. In addition, our protocol establishes specific treatment (Fig. 1) considering immunohistology and microbiology findings on EMB:

- If inflammation is demonstrated with negative viral PCR, treatment with steroids and mycophenolate is indicated for 6 months.
- When inflammation is demonstrated and a positive viral PCR is isolated, treatment with steroids and specific antiviral therapy is prescribed. In the case of myocardial PCR result positive for Parvovirus PVB19, enterovirus or adenovirus, IFN-β is indicated, according to previous literature [13–15].

Despite the use of azathioprine in the TIMIC study [11], we decided to replace it with mycophenolate because it has a better safety profile and similar effects.

A cardiac MRI was performed in hemodynamically stable patients.

Patients were classified based on treatment response. Complete recovery was defined as LVEF ≥ 55% and LEDVz-score ⩽ 2; partial response was defined as those patients with clinical improvement and an LVEF increase of at least 20%; and no response refers to patients who do not show any improvement.

### Statistical analysis

Statistical analysis was performed using SPSS for Windows version 25.0 (Armonk, NY, USA. IBM Corp.). Nominal data were described using proportions and continuous quantitative data employing medians and interquartile range (Q1-Q3) as the sample did not present a normal distribution. Comparisons were made using Fisher’s exact test for categorical data and U-Mann Whitney test for continuous variables where appropriate.

## Results

From February 2015 to December 2024, a cohort of 23 paediatric patients newly diagnosed with inflammatory DCM and severe left ventricular dysfunction (LVEF < 35%) were treated based on the findings from EMB (Table 1). Two additional patients presented with severe left ventricular dysfunction and myocarditis was suspected but EMB was not performed as there were both neonates.

**Table 1:** Treatment outcomes of 23 pediatric patients with newly diagnosed inflammatory dilated cardiomyopathy and severe left ventricular dysfunction

**P**	**E**	**G**	**Age**	**W**	**EF**	**CP**	**ECMO**	**EMB**				
								**Time to EMB**	**Lim T**	**Necrosis**	**Fibrosis**	**Edema**
**1**	**1**	M	27	11,5	32	HF	-	33	80	+ +	-	+
**2**	**2**	F	36	12	25	CS	-	79	50	-	+	+
**3**	**3**	M	26	13	20	CS	17	0	20	-	+	-
**4**	**4**	M	10	9	20	CS	10	12	25	-	+	+
	**5**	M	21	12	31	CS	-	6	15	-	+	-
**5**	**6**	M	7	7,8	21	HF	-	15	50	-	-	+
**6**	**7**	F	10	7,8	23	CS	-	17	70	+	-	+
**7**	**8**	F	39	12,5	23	HF	-	107	180	+	-	+
**8**	**9**	F	9	9	20	CS	8	4	150	-	-	+ +
**9**	**10**	F	33	11	25	HF	-	34	212	+	-	+
**10**	**11**	F	30	13	22	HF	-	10	65	+	-	+
**11**	**12**	M	5	6,8	29	CS	-	24	82	+ +	+	+
**12**	**13**	M	8	7,2	30	CS	7	1	65	+ +	+	+ +
**13**	**14**	M	16	11,5	30	HF	-	62	205	+ +	-	+
**14**	**15**	M	20	10	24	HF	-	6	80	+	-	+
**15**	**16**	F	30	14,6	11	HF	-	15	50	-	-	-
**16**	**17**	F	26	12	17	HF	-	49	18	-	+	-
**17**	**18**	F	11	12,4	18	HF	27	43	20	-	+	-
**18**	**19**	M	158	63	22	HF	22	7	8	-	+	-
**19**	**20**	M	30	11	16	CS	34	19	22	-	-	+
**20**	**21**	F	11	8	34	CS	-	18	11	-	-	-
**21**	**22**	F	6	7,5	17	HF	-	22	9	-	-	+
**22**	**23**	M	2	6,6	25	CS	-	45	23	-	-	+
**23**	**24**	F	110	40	16	HF	78	2	10	-	-	-

**P**	**PCR EMB**	**Treatment**	**Trop**	**Genetics**	**Treatment response**	**Outcome**	**Time to recovery**
**1**	PVB19	CS + IFN β	9,3	-	**Complete**	**Recovery**	212
**2**	PVB19	CS + IFN β	450	-	**Complete**	**Recovery**	241
**3**	-	CS + MMF	12,5		**Complete**	**Recovery**	10
**4**	PVB19 + VH6	CS + IFN β	175	-	**Complete**	**Recovery**	81
	PVB19	CS + IFN β	3,5	-	**Complete**	**Recovery**	8
**5**	-	CS + MMF	2,6	-	**Complete**	**Recovery**	218
**6**	PVB19	CS + IFN β	8,7	-	**Complete**	**Recovery**	184
**7**	PVB19	CS + IFN β	6,9	-	**Complete**	**Recovery**	155
**8**	PVB19	CS + IFN β	5,9	-	**Complete**	**Recovery**	7
**9**	PVB19	CS + IFN β	11,4	-	**Complete**	**Recovery**	56
**10**	PVB19	CS + IFN β	5,6	-	**Complete**	**Recovery**	59
**11**	PVB19	CS + IFN β	10,7	-	**Complete**	**Recovery**	90
**12**	PVB19	CS + IFN β	145,6	-	**Complete**	**Recovery**	13
**13**	PVB19 + VH6	CS + IFN β	613	-	**Complete**	**Recovery**	45
**14**	PVB19	CS + IFN β	7,24	-	**Complete**	**Recovery**	83
**15**	-	CS + MMF	1,9	-	**Improve**	**HF**	-
**16**	-	CS + MMF	1,5	MYH7 (c.2710C > T)	**Improve**	**Tx**	-
**17**	-	CS	3,1	SDHA (p.Arg554Trp p.Lys517Glu)	**Improve**	**HF**	-
**18**	-	CS + MMF	0,4	-	**Improve**	**HF**	-
**19**	-	CS + MMF	5,3	TPM1 (c.62G > C)	**Improve**	**Tx**	-
**20**	PVB19	CS + IFN β	0,15	TNNI3 (c.424G > A)	**No response**	**Tx**	-
**21**	-	CS + MMF	20	FLNC (c.510del)	**No response**	**Tx**	-
**22**	-	CS + MMF	1,7	RPL3L (c.922G > A, c.970G > A)	**No response**	**Tx**	-
**23**	-	CS + MMF	17,5	TNNT2	**No response**	**Tx**	-

*P* Patient, *E* Episodes, *G* Gender, *W* Weight (kg), *EF* ejection fraction (%), *CP* clinical presentation, *HF* heart failure, *CS* cardiogenic shock, *ECMO* extracorporeal membrane oxygenation (days), *EMB* endomyocardial biopsy, *Time to EMB* days between diagnosis and EMB, *Lim T* lymphocyte T/mm^2^, *PCR EMB* viral PCR in endomyocardial biopsy, *PVB19* Parvovirus B19, *PCR blood* Viral PCR in blood test, *CS* corticoesteroids, *IFN β* interferon β, *MMF* mycophenolate, *Trop* troponins (elevation from upper limit), *HF* Heart Failure, *Tx* Transplant, *Time to recovery* days between treatment and recovery

One patient (patient 4) experienced two episodes. The first involved cardiogenic shock requiring ECMO, from which the patient fully recovered after treatment. The second episode occurred one year later, also followed by a complete recovery (Episodes 5 and 6). Of the 23 patients, 12 were females (12/23, 52%), median age at presentation was 20.5 months (IQ 9.3–30) and median weight was 11.3 kg (IQ 7.9–12.5). A single complication related to EMB was registered: a right ventricular free wall perforation in the youngest patient (patient 23:2 months, 8 kg), requiring urgent cardiac surgery and subsequent VA-ECMO support. Median time from diagnosis to EMB was 17.5 days (IQ 6.3–40.8). After a median follow-up of 61 months (IQ: 10.5–94), there were 15 completely recovered patients, 5 patients with partial response to treatment and 4 patients who did not respond to treatment and required cardiac transplant.

Regarding clinical presentation, 13/24 (54.2%) episodes manifested with heart failure symptoms and 11/24 (45.8%) episodes presented with cardiogenic shock. 8/24 (33.3%) episodes required ECMO with a median time of support of 19.5 days (IQR 8.5–32.3). Infectious diseases with fever preceded the majority of episodes (16/24, 66.6%). In terms of family history, none of the patients has a first degree relative affected with DCM.

Microbiological analysis of myocardial tissue revealed a positive PVB19 PCR result in 14 of 24 episodes (58.3%), with two cases showing co-infection with Herpes 6. In all patients with positive PVB19-PCR in cardiac tissue, blood PVB19-PCR was also positive; no cases were observed in which the blood PCR was positive while the endomyocardial biopsy (EMB) result was negative. Myocardial PCR was negative in 10 episodes (41.7%), although respiratory samples from two patients tested positive for COVID-19 (patient 7) and respiratory syncytial virus (RSV) (patient 4).

In relation to genetic testing, 7 out of the 23 patients (30.4%) had pathogenic or likely-pathogenic variants in cardiomyopathic genes (Table 1).

Regarding side effects of medication, there was no severe neutropenia (< 1000neutrophyls/L) in any patient and no infections requiring admission were observed in patients receiving anti- inflammatory treatment. All patients received prophylaxis treatment with trimethoprim/sulfamethoxazole. Mycophenolate blood levels were monitored during treatment, with doses adjusted to maintain levels between 2–4 µg/mL. No severe gastrointestinal adverse effects were reported. Interferon-beta (IFN-β) was well tolerated by all patients except one, who experienced abdominal pain, nausea, and significant elevation of liver enzymes (SGOT 500 UI/L, SGPT 150 UI/L) and bilirubin (4 mg/dL). Treatment was temporarily discontinued for one week and then resumed at half dose following complete symptom resolution. Mild transient elevations of transaminases (up to twice the upper limit of normal) were noted in four other asymptomatic patients and normalized spontaneously without intervention. Fever occurred in two patients after the first administration. No other adverse effects were observed.

Patients were classified regarding treatment response in two groups: group 1, those who present a complete recovery after treatment (15 episodes in 14 patients), and group 2 those with no complete recovery after treatment (9 patients). Of those patients in group 1, full recovery was achieved with a median time of 81 days (IQR 13–184). Of those patients who did not recover completely (group 2), 5 patients had an initial improvement and responded partially to treatment, and 4 patients did not have any improvement and required heart transplant. Among the 5 partially responsive patients, 3 were successfully weaned off ECMO, and all five were eventually discharged home. Two of these patients were listed for transplant at 3 and 1.5 years after discharge, respectively.

Regarding the analysis data, there were no statistical differences comparing both groups (complete recovery vs. no recovery) in terms of age or sex (Table 2). Similarly, clinical presentation, ECMO requirement, and preceding febrile illness were not associated with treatment response. Regarding laboratory findings, median elevation of troponins was 9.3 times the upper limit of normal in group 1 and 1.9 times in group 2 (p 0.015). No significant differences were observed between groups in natriuretic peptide levels. Echocardiographic parameters also showed no statistically significant differences between the two groups across the evaluated measures (Table 2).

**Table 2:** Clinical characteristics of patients with complete response to treatment compared with patients with partial or no response to treatment. (Median, IQR)

	Complete response to treatment (*n* = 15)	Partial or no response to treatment (*n* = 9)	Statistical significance
Age (months)	20 (9–30)	26 (8.5—70)	*p* = 0.64
Gender (female)	40% (6)	66.7% (6)	*p* = 0.4
Weight (kg)	11 (7.8–12)	12 (7.6–27.3)	*p* = 0.41
Clinical presentation (Heart Failure)	46.7% (7)	66.7% (6)	*p* = 0.42
ECMO	26.7% (4)	44.4% (4)	*p* = 0.41
**Blood test**			
Troponin elevation	9.3 (5.9–13.6)	1.9 (1–11.4)	*p*** = 0.015**
Pro-BNP (pg/ml)	23,080 (2860.8–37,188.8)	30,087 (8657–60,369)	*p* = 0.33
**Echocardiogram**			
LVEDD Z-score	+ 4.8 DS (+ 4.5- + 6.1)	+ 5.3 DS (+ 3,5—+ 8,4)	*p* = 0.72
LV hypertrophy	60% (9)	22.2% (2)	*p* = 0.11
MR (moderate to sevre)	60% (9)	66.7% (6)	*p* = 1
LA dilatation (moderate to severe)	80% (12)	44.4% (4)	*p* = 0.1
E/E’ mitral	5 (3–7)	5 (3.5–10)	*p* = 0.73
E/E’ septal	5 (4.3–8.3)	5 (5—8.3)	*p* = 0.64
**EMB**			
CD3/mm2	70 (50–150)	18 (9.5–22.5)	***p* < 0.001**
Edema	80% (12)	33.3% (3)	***p* = 0.036**
Necrosis	60% (9)	0% (0)	***p* = 0.007**
Fibrosis	46.7% (7)	33.3% (3)	*p* = 0.678
Heart PCR	86.7% (13)	11.1% (1)	***p* < 0.001**
**Genetic test**			
Positive genetic test	0% (0)	77.8% (7)	***p* < 0.001**
**Time to treatment**			
Time between diagnosis and EMB/treatment (days)	15 (6–34)	19 (11–44)	*p* = 0.56

*ECMO* extracorporeal membrane oxygenation, *pro-BNP* pro-brain natriuretic peptide, *PCR* polymerase chain reaction, *LVEDD* left ventricular diastolic diameter, *LV* left ventricle, *MR* mitral regurgitation, *LA* left atrial

EMB findings revealed notable differences between the two groups (Table 2). Group 1 demonstrated significantly more intense inflammatory infiltrates compared to group 2 (*p* < 0.001), along with a higher incidence of necrosis and oedema (p 0.007 and p 0.036 respectively). In contrast, Group 2 exhibited milder inflammation, with no cases of necrosis, meeting the diagnostic criteria for inflammatory cardiomyopathy or borderline myocarditis. Additionally, myocardial viral PCR was positive in 86.7% of episodes in Group 1, compared to only 11.1% in Group 2 (*p* < 0.001). No significant differences were observed between groups regarding myocardial fibrosis (p 0.678). Similarly, no statistically significant differences were found regarding the time from diagnosis to EMB performance and treatment initiation (*p* = 0.56).

All patients in Group 1 had negative genetic testing. In contrast, 7 out of 9 patients (77.7%) in Group 2 had a pathogenic or likely pathogenic variants associated with DCM (*p* < 0.001).

## Discussion

We described 24 episodes in 23 patients, presenting with inflammatory DCM and severe left ventricular dysfunction (LVEF < 35%).

In accordance with our protocol, treatment was guided by the results of EMB. Patients were divided into two groups based on their treatment response: those who achieved complete recovery during the follow-up (group 1) and those who exhibited either no response or only partial improvement (group 2).

Regarding the aetiology, a high incidence of pathogenic variants has been reported in patients with myocarditis and inflammatory cardiomyopathy [5, 6]. Some authors have proposed that inflammatory cardiomyopathy may result from ongoing inflammation triggered by a viral infection, in a genetically predisposed individual. Conversely, it is well-established that heart failure triggers an inflammatory process. Indeed, certain types of inflammation are often present in the myocardium of patients with heart failure of diverse aetiologies, including genetic cardiomyopathy [8–10]. In these cases, inflammation is characterized by no massive tissue injury and limited inflammatory activity mainly mediated by tissue‐resident cells, and is known as “para‐ inflammation”, or “low-grade (smouldering) chronic inflammation” [20].

In our cohort, we observed some differences between patients who achieved complete recovery (group 1) and those who did not (group 2):

Group 1 (Table 2) exhibited the absence of pathogenic variants and 86.7% had positive myocardial viral PCR. Troponin levels were higher in these patients (showing more inflammation) and EMB revealed a higher number of inflammatory cells and higher prevalence of myocardial oedema and necrosis.

These findings suggest that patients in group 1, were previously healthy individuals, experiencing a viral-induced myocarditis. Ongoing inflammation, with or without persistent viral replication, may have subsequently contributed to the development of DCM. Effective treatment targeting both inflammation and persistent viral infection, when present, may improve patient outcomes and facilitate complete recovery of cardiac function. Regarding viral aetiology, PVB19 was found alone or in co-infection with HHV6, suggesting that in paediatric patients, PVB19 is the leading cause of myocarditis.

Conversely, in group 2, viral PCRs were negative in 88.9%, there was a low grade of inflammation, necrosis was absent and a pathogenic variant was found in 77.8%. These findings suggest that most of these patients were affected with new-onset DCM of genetic origin. In these cases, the observed low-grade inflammation may be a secondary consequence of the hemodynamic and neurohormonal alterations associated with heart failure [20]. While complete recovery was not achieved in any of these patients, partial responses to treatment were observed in some of them, and heart transplantation could be avoided or deferred for several months or years.

Interestingly, there were no significant differences between groups regarding the presence of fever before to the episode. Paediatric patients often experience multiple infections during early childhood, and an infection could act both as the underlying cause of the event or as a trigger for cardiac destabilization in a patient with pre-existing severe dysfunction.

Regarding treatment, in patients with inflammatory cardiomyopathy and a negative viral PCR in the myocardium, immunosuppressive therapy has been described as a potential treatment option [4, 10, 11]. In our protocol, immunosuppression comprised a combination of corticosteroids and mycophenolate. In cases where viral PCR was positive, antiviral therapy was added to steroids. Our experience treating patients with PVB19-myocarditis, with IFNꞵ and steroids has been previously described [17]*.* Remarkably, all patients with a positive PVB19-PCR in cardiac tissue also had a positive blood PVB19-PCR. This finding may have important implications for clinical management. In very young patients (< 7 kg) for whom EMB is considered of high risk, the combination of severe ventricular dysfunction, a positive blood PVB19-PCR, and markedly elevated troponin levels could support the early initiation of treatment. Future studies in larger cohorts may help define troponin cut-off values for clinical decision-making.

Interestingly, no statistically significant differences were observed between both groups in the time between diagnosis and EMB performance and treatment initiation (p 0.56). This is a notable finding, as some patients who achieved complete recovery had been diagnosed with severe dysfunction several weeks before EMB was performed and treatment initiated (most of them referred from other centres). Yet, the time in which the EMB was performed did not correlate with worse outcomes in our series.

Frustaci et al. [21] sought to identify factors to predict a patient's response to immunosuppression, concluding that it could be helpful for those patients with a negative PCR in myocardium. Contemporary diagnostic approaches now encompass a broader range of tools, including genetic testing and histopathological evaluation that may help refine case selection and guide assessment of treatment response.

In a study [6], pathogenic or likely-pathogenic variants were identified in 7 out of 20 (35%) paediatric patients with DCM secondary to myocarditis. Only 30% of the patients achieved complete recovery, and 40% died or required heart transplantation. In comparison, 7 out of 23 (30.4%) paediatric patients in our cohort with inflammatory DCM presented pathogenic or likely pathogenic variants. Twenty-four episodes were treated based on the results of EMB, and complete recovery was achieved in 62.5% of them.

Our study suggests that a subset of patients with inflammatory cardiomyopathy could benefit from EMB-guided treatment. In our view, the most strongly related factor to predict the response to treatment, is the underlying cause. Those patients with viral myocarditis, with or without persistent infection, respond better than those with a suspected genetic cardiomyopathy. While awaiting the results of genetic testing, which may require several weeks or months, other factors can add some information to predict the response. Notably, a positive viral PCR in myocardium and the presence of significant inflammation, oedema and necrosis, may potentially indicate a favourable response to treatment. While we cannot exclude the possibility that some of our patients may have recovered without treatment, our experience suggests this therapeutic approach could improve the outcome for these patients. This assertion can only be definitively supported by a randomized controlled trial.

The clinical significance of a positive PVB19 PCR in the myocardium in adult patients remains unclear and is currently under debate [22, 23]. Viral DNA copy number has been proposed as a tool to differentiate between active and latent infection [23]. However, in our experience, in paediatric patients, particularly younger ones, the detection of viral genome in blood and myocardial tissue strongly suggests a viral aetiology for inflammatory cardiomyopathy, but further studies are needed to evaluate methods to differentiate between previous and current PVB19 and HHV6 infection in paediatric patients.

## Conclusion

To our knowledge, this is the first paediatric study of patients presenting with DCM in which EMB is performed, myocardial inflammation is demonstrated and treatment is prescribed regarding histologic and microbiological results. A significant proportion of these patients have pathogenic or likely pathogenic variants in cardiomyopathic genes. Furthermore, our results suggest that the main factor predicting a patient's response to treatment is the presence of pathogenic or likely pathogenic variants. Consequently, we believe, genetic study should be performed om these patients. Other important factors associated with a favourable response to treatment include intense inflammatory infiltrate in EMB, oedema, necrosis, and a positive viral PCR in the myocardium. Prospective and multicentre studies are needed to corroborate these findings.

## Study limitations

This is a case series observational study and has inherent limitations, including the absence of a control group and randomization. In addition, the limited sample size, inherent to the rarity of this condition, precludes drawing definitive conclusions about the superiority of the proposed treatment regimen compared to standard care alone. More studies are needed to evaluate these findings.

## Data Availability

No datasets were generated or analysed during the current study.
